# Toxic epidermal necrolysis caused by phenobarbital: a case report and literature review

**DOI:** 10.3389/fphar.2024.1433506

**Published:** 2024-08-01

**Authors:** Jie Cheng, Hui Li, Yan Li, Xiao Li, Jianjun Wang, Xin Huang, XueYan Cui

**Affiliations:** ^1^ Department of Clinical Pharmacy, The First Affiliated Hospital of Shandong First Medical University and Shandong Provincial Qianfoshan Hospital, Shandong Medicine and Health Key Laboratory of Clinical Pharmacy, Jinan, China; ^2^ Department of Clinical Pharmacy Laboratory, The First Affiliated Hospital of Baotou Medical College, Baotou, China; ^3^ Department of Neurology, Fei Xian People’s Hospital, Linyi, China; ^4^ Department of Neurosurgery, The First Affiliated Hospital of Shandong First Medical University and Shandong Provincial Qianfoshan Hospital, Shandong Medicine and Health Key Laboratory of Neurosurgery, Jinan, China

**Keywords:** phenobarbitone, toxic epidermal necrosis, CYP2C19∗1/∗2, HLA-B∗15:02, HLA-B∗58:01

## Abstract

**Background:**

Toxic epidermal necrolysis (TEN) and Stevens-Johnson syndrome (SJS) are rare, life-threatening immunologic reactions. Previous relevant literature has provided limited information regarding this disease’s genetic susceptibility and management principles.

**Objectives:**

This study aimed to describe a phenobarbital-induced TEN case report with HLA-B*15:02 and HLA-B*58:01 negative, CYP2C19*1/*2. In addition, we revised the existing literature on phenobarbital-induced SJS/TEN to explore its clinical characteristics.

**Methods:**

We describe a woman undergoing treatment with Phenobarbital for status epilepticus who developed classic cutaneous findings of TEN. A systematic search was conducted in the PubMed, Medline, WanFang, and CNKI databases from 1995 to 2023. The search terms used were “Stevens-Johnson Syndrome,” “Toxic Epidermal Necrolysis,” and “Phenobarbital.”

**Results:**

We report a case of TEN resulting from phenobarbital; it tested negative for the HLA-B*15:02 and HLA-B*58:01 allele and CYP2C19*1/*2 intermediate metabolism. Supportive treatment with steroids and antihistamines resulted in complete resolution of the skin lesions and improvement in clinical symptoms after 14 days. Physicians and clinical pharmacists should be aware of these potential phenobarbital-related adverse events and closely monitor patients with first-time use of phenobarbital. Among 19 cases were identified in the literature, with 11 (57.9%) cases of SJS, 6 (31.6%) cases of TEN, and 2 (7.2%) cases of SJS-TEN/DRESS overlap. A total of 5 (26.3%) did not survive, of which 4 (21.1%) were under 12 years old and 1 (5.3%) was over 12 years old.

**Conclusion:**

Phenobarbital-induced SJS/TEN may still occur in patients who test negative for HLA-B*15:02 and HLA-B*58:01, CYP2C19*1/*2. Most cutaneous adverse events occur early in the course of Phenobarbital therapy and should be closely monitored early in the course of treatment. In addition, Phenobarbital should be used with caution in patients with a history of asthma and allergy to antipyretics and analgesics.

## 1 Introduction

Toxic epidermal necrolysis (TEN) and Stevens-Johnson syndrome (SJS) are potentially life-threatening type IV hypersensitivity reactions that present with mucocutaneous blistering reactions with epidermal detachment and extensive necrosis, with an estimated incidence of 1.1–6.0 per million (Valeyrie-Allanore and Roujeau, 2012; White et al., 2018). Although with a low incidence, SJS and TEN can result in disability or death with a mortality ranging from 10% to 40% (Yang et al., 2011). Phenobarbital (PB), along with other aromatic antiepileptic drugs (AEDs), such as carbamazepine (CBZ) and phenytoin (PHT), is known to cause hypersensitivity reactions ranging from 1 to 63 days after starting treatment (Mockenhaupt et al., 2004; Trivedi et al., 2017; Borrelli et al., 2018). The risk factors of TEN/SJS include a history of allergy, advanced age, HIV infection, pre-existing liver disease, and chronic underlying diseases (Lerch et al., 2018). Recent studies have shown that different ethnic populations may have dissimilar risks regarding developing AED-induced adverse reactions due to various genetic backgrounds (Yang et al., 2011). Chinese Han carriers of the HLA-B*15:02 allele have a strong genetic association with carbamazepine-induced SJS/TEN (Chung et al., 2004; Man et al., 2007). The US Food and Drug Administration updated the CBZ label to include genetic information on the HLAB*15:02 allele. It recommended genetic screening for the HLA-B*15:02 allele in all patients of Asian ethnicity with epilepsy before starting CBZ therapy (Ferrell and McLeod, 2008).

Furthermore, it has been demonstrated that the correlation between CBZ-induced SJS/TEN and the HLA-B*15:02 allele extends to other aromatic AEDs, such as PB-associated SJS/TEN in the Chinese Han population (Sun et al., 2014). Genetic polymorphisms of *CYP2C19* contribute to the pharmacokinetic variability of phenytoin and phenobarbital, the poor metabolizers of *CYP2C19*, which are relatively common in Asian groups (Yukawa and Mamiya, 2006; Goto et al., 2007). The most common variants resulting in poor metabolizers are CYP2C19*2/*3 (Chen, 2014). The plasma concentration of phenobarbital is elevated in individuals carrying CYP2C19*2 and CYP2C9*3 gene variants (Qi, 2011). However, the impact of *CYP2C19* gene polymorphisms on severe cutaneous adverse reactions (SCARs) induced by aromatic anticonvulsants is still unclear. This report presents a case of PB-induced TEN in the Chinese Han population who tested negative for the HLA-B*15:02 and HLA-B*58:01 allele but CYP2C19*1/*2 intermediate metabolism.

## 2 Materials and methods

### 2.1 Case description

**FIGURE 1:**
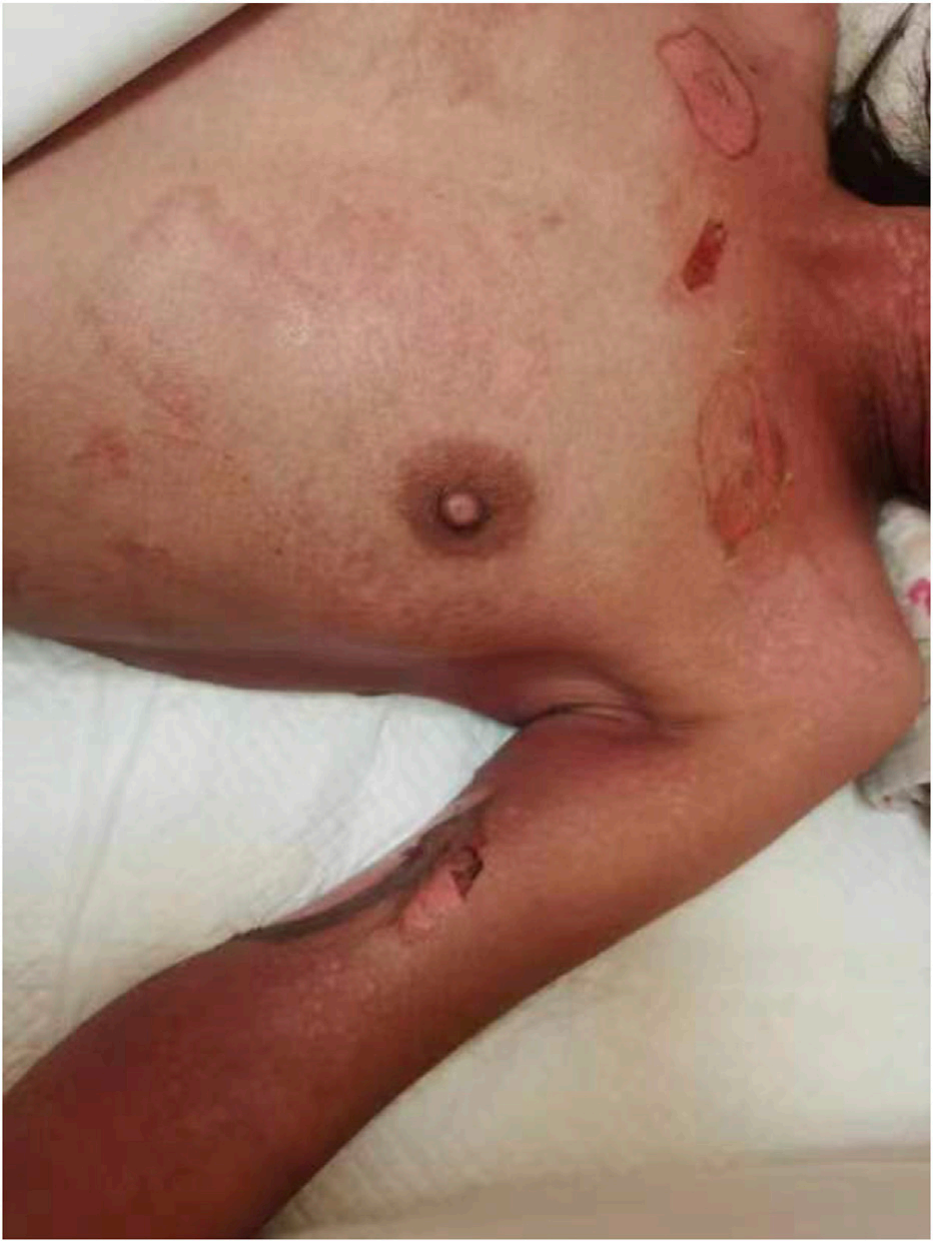
Clinical Findings of Toxic epidermal necrolysis.

A 38-year-old Chinese woman was admitted to our Neurosurgery department due to status epilepticus (SE) on 26 July 2023. The patient had a 20-year history of generalized tonic-clonic seizures (GTCS) and was treated with sodium valproate (VPA), levetiracetam (LEV), and lacosamide (LCM). After admission, the patient was treated with antiepileptic drugs, including intramuscular phenobarbital 100 mg bid (only used five times), intravenous levetiracetam 500 mg bid, and midazolam. In addition, the patient continued to receive oral sodium valproate at 100 mg bid and lacosamide at 50 mg bid. On the second day, the patient began to experience fever, up to T38.9°C, without sore throat or cough, and the pharmacist recommended checking for infectious indicators. On the third day, the patient still had a fever, but the infection indicators, such as blood routine white blood cell counts and procalcitonin, were standard, and lung CT was standard. The patient’s skin became red, blistered, and peeled extensively over the body, Nikolsky positive (Figure 1). Phenobarbital was administered only 5 times, and blood was taken 10 h after the last dose to measure the concentration. The blood concentration of the antiepileptic drug was determined using the Liquid chromatography-mass spectrometry (LC-MS) method. The blood concentration tests revealed that the levels of phenobarbital (12.512 μg/mL), valproic acid (72.823 μg/mL), levetiracetam (24.812 μg/mL), and lacosamide (4.396 μg/mL) were within the therapeutic reference range. After a multidisciplinary team (MDT) with a Dermatologist, Clinical pharmacist, and Infection physician, it is considered that toxic epidermal necrolysis (TEN) caused by phenobarbital, and it is recommended to provide the patient with dexamethasone, anti-allergic treatment, and treatment such as using comfrey oil to scrape and break the skin. Discontinue all drugs except midazolam, dexamethasone, and loratadine were given to mitigate the allergy. According to the clinical pharmacist’s advice, risk factors for developing this allergy were determined, including phenobarbital blood levels and associated risk genes HLA-B*15:02 and HLA-B*58:01, as well as *CYP2C19*. In terms of patient care, the clinical pharmacist suggests that to prevent and control infection, the topical drug comfrey oil should be applied with sterile cotton balls, and the sterile sheet should be changed at least once daily.

Subsequently, the patient was admitted to the Department of Dermatology. The estimated skin detachment was approximately 30% of the body surface area. After written informed consent was obtained, *HLA-B* and *CYP2C19* genetic testing was carried out by Fluorescence *In Situ* Hybridization (FISH) method. On the sixth day of admission, the patient’s *HLA* high-resolution genotyping showed a negative result for the HLA-B*58:01 and HLA-B *15:02 allele. Furthermore, the *CYP2C19* high-resolution genotyping indicated that the patient is a *CYP2C19* intermediate metabolizer with CYP2C19*1/*2.

The causality assessment of the reaction was done using the Naranjo scale (Naranjo et al., 1981). The causality of phenobarbitone in this reaction was 6 points on the Naranjo scale; the interpretation of the scores was probable. The patient prognosis assessment used the SCORTEN scoring system (Fouchard et al., 2000). The patient mortality rate was estimated to be 12% in this case. On subsequent days in Dermatology, the patient’s clinical condition improved; the skin lesion started healing and exfoliating in most affected skin surface areas. The clinical pharmacist performs antiepileptic medication reconciliation based on changes in the patient’s condition and collaborates with the supervising physician to develop a plan. On the 10th day, the clinical pharmacist recommended restarting the patient’s oral antiepileptic medication, which included sodium valproate, levetiracetam, and perampanel. After receiving 14 days of treatment with steroids and antihistamines, the patient’s epileptic symptoms were under control, and skin allergies did not reappear.

## 3 Literature review

### 3.1 Search strategy

We searched PubMed, Medline, WanFang, and CNKI databases to retrieve case reports on phenobarbital-induced SJS/TEN published between 1 January 1995 and 31 December 2023. The search terms used were combined text and Medical Subject Headings (MeSH) search strategy was used to search the above databases: (Stevens-Johnson Syndrome OR Toxic Epidermal Necrolysis) AND (Phenobarbital). An equivalent translation of the same search terms was used to search Chinese databases. We considered only case reports and excluded original research, surveys, conference abstracts, editorials, and reviews. This study adhered to the guidelines outlined by the Preferred Reporting Items for Systematic Reviews and Meta-Analyses (PRISMA) (Page et al., 2021a; 2021b).

### 3.2 Inclusion and exclusion criteria

Among all of the case reports that described associations with phenobarbital-induced SJS/TEN associations, we applied the following eligibility criteria: 1) SJS/TEN diagnosed on clinical and/or histopathologic criteria (Schwartz et al., 2013; McPherson et al., 2019); 2) sufficient description of Phenobarbital is associated with SJS/TEN; 3) specific information on Phenobarbital triggers provided; 4) enough details about the acute phase of the SJS/TEN.

### 3.3 Data collection

**FIGURE 2:**
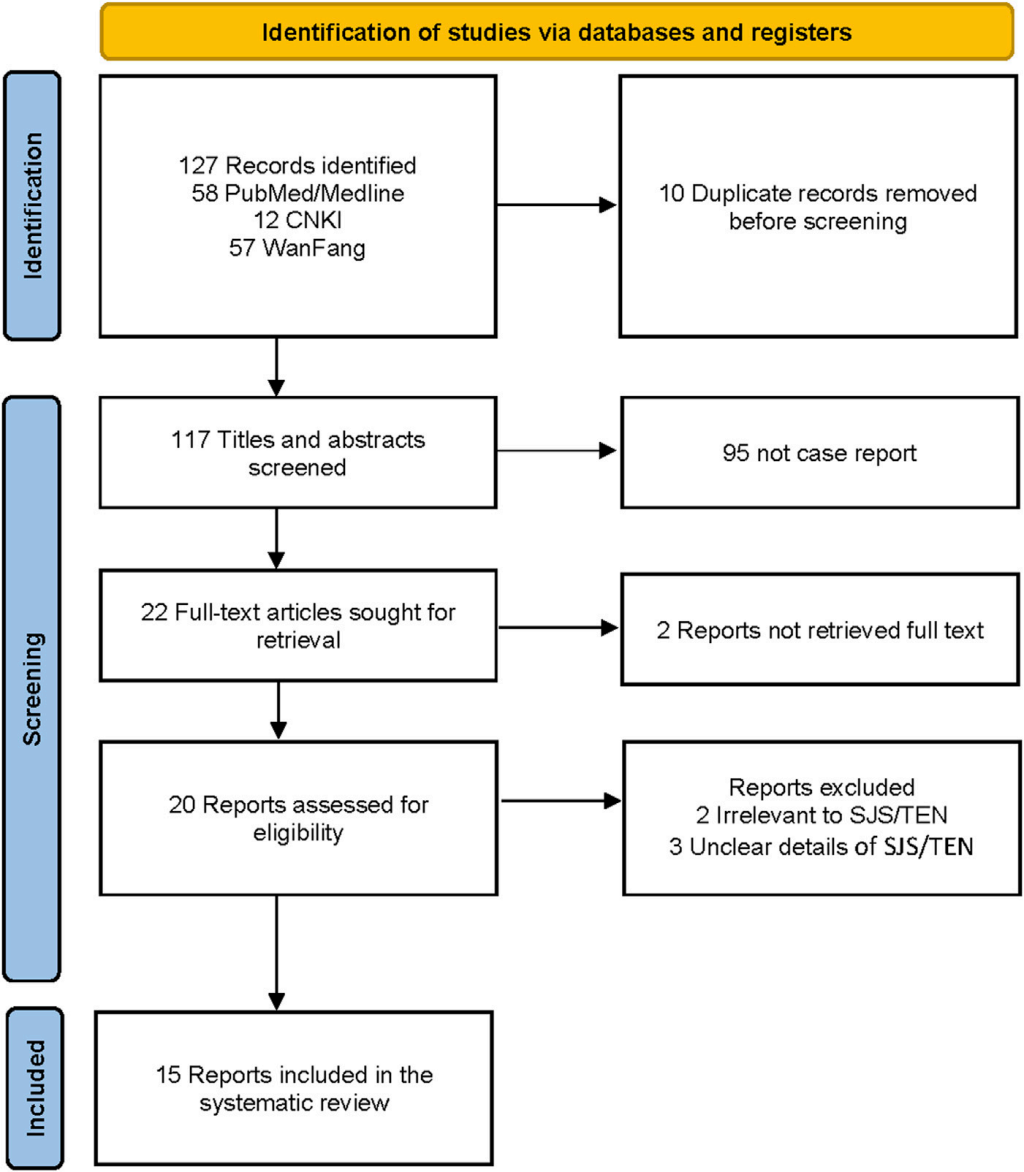
PRISMA flowchart for selecting phenobarbital-induced SJS/TEN case reports for analysis.

Two researchers independently screened records and extracted data added to a dedicated spreadsheet. Any conflict between the two researchers was resolved by reaching a consensus or by a third reviewer. The PRISMA flowchart diagram summarized the literature search and final study selection (Figure 2). The following information was extracted from the reports using a pre-defined data extraction template. Report characteristics included publication year and country. Patient information included gender, age, race, time of adverse reaction, genetic test results, management principle, length of stay (LOS), history, outcome, Phenobarbitone dose, and SCAR type.

## 4 Results

### 4.1 Study selection

Figure 2 shows the PRISMA diagram for selecting case reports to be included in the analysis (Page et al., 2021b; Page et al., 2021a). The literature search resulted in 127 records, of which 10 were duplicates and removed. We excluded 95 records that were not case reports, 2 reports without full text, 2 irrelevant to SJS/TEN, and 3 with unclear details of SJS/TEN. The literature review included 15 case reports (Zhang, 1995; Duncan et al., 1999; Hu, 2001; Zhou, 2003; Qiu, 2005; Wei, 2006; Agnihotri and Gaur, 2012; Kheir and Hamed, 2012; Li, 2013; Kaputu-Kalala-Malu et al., 2014; Jing, 2018; Zankat et al., 2018; Ayele et al., 2021; Singh et al., 2022; Van Nguyen et al., 2023) with 18 patients from 7 countries.

### 4.2 Clinical characteristics of phenobarbital-induced SJS/TEN

In this study, amalgamated with our current case, 19 patients were included. Among 19 cases, there were 11 (57.9%) SJS, 6 (31.6%) TEN, and 2 (7.2%) SJS-TEN/DRESS overlap cases. The overall demographics and clinical characteristics of these patients are listed in Table 1. Approximately 52.6% (n = 10) of all included cases were female. The age range was from 1 to 74, with a median age of 12. A total of 5 (26.3%) did not survive, of which 4 (21.1%) were under 12 years old and 1 (5.3%) was over 12 years old. Out of all 19 cases, 11 (57.9%) patients had an epilepsy diagnosis and were receiving antiepileptic therapy. Six (31.6%) patients presented with a craniocerebral injury or encephalitis and three (15.8%) patients were diagnosed with asthma or a history of drug allergies. The onset of SCARs varied from 1 to 42 days, with an average of 13.8 days and a median of 14 days. The mean dosage of PB was 145 mg/day, and the mean LOS was 16.5 days. Only three (15.7%) patients were genotyped; one was HLA-B*15:02 positive, and two were HLA-B*15:02 negative.

**TABLE 1:** Clinical characteristic, the genotype of Phenobarbital-induced SJS/TEN.

Patient	Sex	Age (years)	Country	Time of adverse reaction(d)	LOS(d)	Phenobarbitone dose	SCARs type	Genetic test results	History	Outcome
1	Female	1	China	2	14	20 mg tid	TEN	-	Epilepsy	Death
2	Male	3	China	15	-	-	SJS	-	Epilepsy	Death
3	Female	74	China	19	6	100 mg bid	SJS	-	Intracranial Hemorrhage, family Antipyretic allergic history	Cure
4	Female	19	China	3	22	60 mg tid	TEN	-	Epilepsy	Cure
5	Male	13	China	15	15	100 mg bid	SJS	-	Epilepsy	Cure
6	Female	6	China	21	5	-	SJS	-	Epilepsy	Death
7	Female	2	China	5	21	-	SJS	-	Viral encephalitis	Cure
8	Male	12	China	40	3	50 mg tid	SJS	-	Epilepsy	Death
9	Female	31	China	1	14	-	TEN	-	Spin	Cure
10	Female	45	China	8	15	100 mg tid	SJS	HLA-B*15:02 negative, *CYP2C19* intermediate metabolizers	Autoimmune Encephalitis	Death
11	Male	53	American	42	56	200 mg qd	SJS	-	Brain tumour	Cure
12	Male	14	Ethiopia	14	10	100 mg bid	TEN	-	Asthma	Cure
13	Male	4	Vietnam	25	28	-	SJS-Dress overlap	HLA-B*15:02 positive, *CYP2C19* Normal metabolism	Epilepsy	Cure
14	Male	2	Congo	1	25	50 mg qd	TEN	-	Intracranial Haemorrhage	Cure
15	Female	18	India	15	20	30 mg bid	SJS	-	Epilepsy	Cure
16	Female	33	India	13	10	60 mg bid	SJS-TEN overlap	-	Epilepsy	Cure
17	Male	12	India	7	10	60 mg qd	SJS	-	Epilepsy	Cure
18	Male	5	Sudan	14	10	30 mg bid	SJS	-	Epilepsy	Cure
19	Female	38	China	2	14	100 mg bid	TEN	HLA-B*15:02 negative, *CYP2C19* intermediate metabolizers	Epilepsy, Analgesics allergic history	Cure

## 5 Discussion

Based on a retrospective pharmacovigilance study using VigiBase^®^, phenobarbital accounted for 5.7% of the SCARs caused by anti-seizure medicine (ASM) worldwide, and Asian people have a higher risk of phenobarbital-induced SCARs than the world (Shukla et al., 2021). In addition, this study found that phenobarbital has a significant pharmacovigilance signal in drug rash with eosinophilia and systemic symptoms (DRESS) cases (Shukla et al., 2021). A retrospective study based on the Taiwan Drug-Injury Relief Database (TDRD) also shows that phenobarbital was one of the main culprit drugs in DRESS (Huang et al., 2022). Studies from Iran have shown that phenobarbital is the most common culprit drug for SJS/TEN in pediatric patients (Abtahi-Naeini et al., 2022; Abtahi-Naeini et al., 2024). The above studies have demonstrated the close association between phenobarbital and SCARs, and understanding their clinical features is essential for preventing phenobarbital-associated SCARs.

In the present study, we reported a case of phenobarbitone-induced TEN and retrieved several similar cases from the literature. The present case describes a woman who presented with clinical features suggestive of TEN with extensive surface area involvement after exposure to phenobarbitone. The 38-year-old female patient reported in this study met the diagnostic criteria for PB-induced TEN according to Roujeau’s classification (Roujeau, 1994). The incidence of SJS/TEN due to phenobarbital use was 1.3 per 10,000 person-years (Mockenhaupt et al., 2004). To our knowledge, this is the first analysis of the onset of phenobarbital-induced SJS/TEN. The incidence of TEN was 31.6% (n = 6), lower than that of SJS 57.9% (n = 11), which is consistent with the existing literature (Hsu et al., 2016). The mortality of phenobarbital-induced SJS/TEN was 26.3% (n = 5), significantly higher than the 6.1% reported in the literature (Yang et al., 2011). The mortality rate of 44.4% in individuals under 12 years of age is significantly higher than the 10% rate in those over 12 years of age, which is the main reason. The morbidity associated with SJS/TEN in children is relatively low, ranging from 0.4 to 5.3 per million, but the associated mortality is high, ranging from 16.7% to 44% (Hsu et al., 2017; Shi et al., 2020).

After admission, the patient was treated with phenobarbital, levetiracetam, sodium valproate, lacosamide, and midazolam. This patient developed fever on the second day and a rash on the third day of phenobarbital application. For the AEDs known to be associated with these severe reactions, most reactions (>90%) occurred ranging from 1 to 63 days after starting treatment (Mockenhaupt et al., 2004). In our literature review, the onset of SCARs varied from 1 to 42 days, with an average of 13 days. Patients receiving phenobarbital should be monitored closely for possible adverse effects in the initial period.

Genetic testing is a vital tool for identifying the underlying cause of SJS/TEN, which may facilitate the prevention of these reactions in some cases (Alfirevic et al., 2019). However, this study revealed that only three (15.7%) patients underwent genetic monitoring following the onset of SJS/TEN, indicating a need to enhance the management response to SJS/TEN. Human leukocyte antigen (*HLA*) genetic variation is implicated in developing specific cutaneous adverse reactions to aromatic anticonvulsants (Phillips et al., 2018). AED-induced SJS/TEN has been widely reported with solid associations with HLA-B*15:02 and HLA-B*58:01 (Hung et al., 2010; Cheung et al., 2013). The variant alleles HLA-B*15:02 and HLA-B*58:01 are associated with a significant risk of SJS/TEN (Chung et al., 2004; Kaniwa et al., 2008). In the present study, the HLA-B*15:02 and HLA-B*58:01 tests were negative.

It has been demonstrated that the risk of experiencing adverse events increases when the concentration of phenobarbital in the blood is raised (Yasiry and Shorvon, 2012; Sun et al., 2019). However, we have only found one report linking high levels of phenobarbital to induce SJS/TEN (Jing, 2018). Co-administration of valproate with phenobarbital may increase the plasma concentration of phenobarbital (Bourgeois, 1988). The pharmacokinetic variability of AED is influenced by the genetic polymorphisms of *CYP2C19* (Yukawa and Mamiya, 2006). The plasma phenobarbital concentration is higher in CYP2C19*2 and CYP2C9*3 mutation carriers in the Chinese Han population (Qi, 2011). In Thai children carrying the CYP2C19*2 variant and treated with phenobarbital, there was a significant 4.5-fold increase in the risk of developing severe cutaneous adverse reactions (SCARs) compared to the drug-tolerant control group (Manuyakorn et al., 2013). However, a study conducted in the Japanese population found no significant difference in the pharmacokinetic parameters of PB between *CYP2C19* genotypes (Lee et al., 2012). In this case, the *CYP2C19* gene was tested as an intermediate metabolizer, and blood levels were in the therapeutic range this was probably due to the time of blood collection being later than the onset of adverse reactions. Additionally, this case has an analgesics allergic history (paracetamol); allergic patients have a greater risk of developing SJS/TEN (Noe and Micheletti, 2020). Considering the complexity of the SJS/TEN mechanism, it remains to be investigated the relationship between SJS/TEN, phenobarbital blood level, and *CYP2C19* gene variants. Multidisciplinary care in toxic epidermal necrolysis includes identifying the culprit drug, supportive medical care, nutritional support, physical and occupational therapy, and genetic assessment of susceptibility to SJS/TEN. Having a clinical pharmacist involved in patient medication and adverse reaction monitoring can significantly improve patient prognosis (Cheng et al., 2023). The most crucial measure in the acute phase is the immediate withdrawal of the culprit drug when there is suspicion of SJS/TEN (Shanbhag et al., 2020). This patient developed blisters or erosions after taking antiepileptic medication. The clinical pharmacist promptly identified this adverse reaction, discontinued the suspected drug, and administered anti-allergy treatment. Supportive care encompasses protecting and restoring the skin’s barrier function, maintaining fluid balance, protecting the airway, and treating infection (Saeed et al., 2016). The clinical pharmacist assisted the nurses with patient wound care and timely detection of contaminated linens to avoid secondary infection of the patient’s skin tissue. Measuring drug concentrations and associated risk genes is crucial for identifying suspect drugs and developing antiepileptic therapy. The clinical pharmacist identified phenobarbital as the suspected drug causing TEN. Clinical pharmacists significantly improve patient outcomes by participating in the monitoring of patients’ medication regimens. In a multidisciplinary effort that includes clinical pharmacists, the patient cured and discharged. The average LOS observed in this study was approximately 16.5 days, which was lower to the average LOS of 23.9 days reported in a previous study (Cheung et al., 2024). The incidence of Stevens-Johnson syndrome (SJS) and toxic epidermal necrolysis (TEN) caused by phenobarbital has become a significant global health burden, leading to prolonged LOS and mortality.

There are several limitations to our study. First, this study only tested the HLA-B*15:02, HLA-B*58:01, and *CYP2C19*, the other genes previously reported associated with adverse reaction risk such as HLA-B*15:11 and HLA-A*31:01 were not measured (McCormack et al., 2011; Sun et al., 2014). Second, the fact that blood of concentration was taken approximately 10 h after the last dose. Third, although our literature review included all published literature on phenobarbital-induced SJS/TEN, the sample size was still small. Further study with a larger sample size is necessary to address these limitations.

## 6 Conclusions

This is a case of phenobarbital-induced TEN in an HLA-B*15:02 and HLA-B*58:01 negative and CYP2C19*1/*2 intermediate metabolism patient; unlike previously reported similar cases, the patient’s cutaneous eruption was promptly recognized, and treated appropriately under close monitoring by physicians and clinical pharmacists, and the patients had a good prognosis. In a multidisciplinary effort that includes clinical pharmacists, the patient cured and discharged. Through the literatures review, HLA-B*15:02 and HLA-B*58:01 and the elevated concentration of phenobarbital were associated with increased risk of SJS/TEN. The onset of SCARs varied from 1 to 42 days, with an average of 13.8 days and a median of 14 days. The relationship between SJS/TEN caused by PB and susceptibility genes such as *HLA-B* and *CYP2C19* in different populations requires further evaluation.

## Data Availability

The raw data supporting the conclusions of this article will be made available by the authors, without undue reservation.

## References

[B1] Abtahi-Naeini B. Dehghan M.-S. Paknazar F. Shahmoradi Z. Faghihi G. Sabzghabaee A. M. (2022). Clinical and epidemiological features of patients with drug-induced stevens-johnson syndrome and toxic epidermal necrolysis in Iran: different points of children from adults. Int. J. Pediatr. 2022, 8163588. 10.1155/2022/8163588 35178096 PMC8847037

[B2] Abtahi-Naeini B. Makhmali R. Amini N. Reza Maracy M. Nouri N. Momen T. (2024). Antiepileptic medication-induced severe cutaneous adverse reactions in hospitalized children: a retrospective study. Iran. J. Allergy Asthma Immunol. 23, 139–148. 10.18502/ijaai.v23i2.15320 38822509

[B3] Agnihotri R. Gaur S. (2012). Phenobarbital induced Stevens-Johnson syndrome in a child. Indian J. Pharmacol. 44, 531–532. 10.4103/0253-7613.99344 23087523 PMC3469965

[B4] Alfirevic A. Pirmohamed M. Marinovic B. Harcourt‐Smith L. Jorgensen A. L. Cooper T. E. (2019). Genetic testing for prevention of severe drug‐induced skin rash. Cochrane db. Syst. Rev. 7, CD010891. 10.1002/14651858.CD010891.pub2 PMC6636675 31314143

[B5] Ayele B. A. Ali K. Mulatu E. (2021). Toxic epidermal necrosis associated with phenobarbitone: a case report and brief review of the literatures. Allergy Asthma Clin. Immunol. 17, 88. 10.1186/s13223-021-00589-4 34496964 PMC8425050

[B6] Borrelli E. P. Lee E. Y. Descoteaux A. M. Kogut S. J. Caffrey A. R. (2018). Stevens‐Johnson syndrome and toxic epidermal necrolysis with antiepileptic drugs: an analysis of the US Food and drug administration adverse event reporting system. Epilepsia 59, 2318–2324. 10.1111/epi.14591 30395352 PMC6420776

[B7] Bourgeois B. F. D. (1988). Pharmacologic interactions between valproate and other drugs. Am. J. Med. 84, 29–33. 10.1016/0002-9343(88)90054-X 3146222

[B8] Chen H. (2014). Impact of CYP2C9 and CYP2C19 gene polymorphisms on drug metabolism and advances in individualized dosing research. China Drug Appl. Monit. CNKI:SUN:YWYY.0.2014-04-018.

[B9] Cheng J. Dang C. Li X. Wang J. Huang X. Li Y. (2023). The participation of clinical pharmacists in the treatment of patients with central nervous system infection can improve the effectiveness and appropriateness of anti-infective treatments: a retrospective cohort study. Front. Pharmacol. 14, 1226333. 10.3389/fphar.2023.1226333 37745082 PMC10512419

[B10] Cheung C. M. Chang M. M. Li J. J. Chan A. W. (2024). Stevens–Johnson syndrome and toxic epidermal necrolysis in Hong Kong. Hong Kong Med. J. 30, 102–109. 10.12809/hkmj2210131 38531617

[B11] Cheung Y. Cheng S. Chan E. J. M. Lo S. V. Ng M. H. L. Kwan P. (2013). HLA-B alleles associated with severe cutaneous reactions to antiepileptic drugs in Han Chinese. Epilepsia 54, 1307–1314. 10.1111/epi.12217 23692434

[B12] Chung W.-H. Hung S.-I. Hong H.-S. Hsih M.-S. Yang L.-C. Ho H.-C. (2004). Medical genetics: a marker for Stevens-Johnson syndrome. Nature 428, 486. 10.1038/428486a 15057820

[B13] Duncan K. O. Tigelaar R. E. Bolognia J. L. (1999). Stevens-Johnson syndrome limited to multiple sites of radiation therapy in a patient receiving phenobarbital. J. Am. Acad. Dermatol. 40, 493–496. 10.1016/S0190-9622(99)70508-6 10071329

[B14] Ferrell P. B. McLeod H. L. (2008). Carbamazepine, HLA-B*1502 and risk of Stevens-Johnson syndrome and toxic epidermal necrolysis: US FDA recommendations. Pharmacogenomics 9, 1543–1546. 10.2217/14622416.9.10.1543 18855540 PMC2586963

[B15] Fouchard N. Bertocchi M. Roujeau J.-C. Revuz J. Wolkenstein P. Bastuji-Garin S. (2000). SCORTEN: a severity-of-illness score for toxic epidermal necrolysis. J. Invest. Dermatol. 115, 149–153. 10.1046/j.1523-1747.2000.00061.x 10951229

[B16] Goto S. Seo T. Murata T. Nakada N. Ueda N. Ishitsu T. (2007). Population estimation of the effects of cytochrome P450 2C9 and 2C19 polymorphisms on phenobarbital clearance in Japanese. Ther. Drug Monit. 29, 118–121. 10.1097/FTD.0b013e318030def0 17304159

[B17] Hsu D. Y. Brieva J. Silverberg N. B. Paller A. S. Silverberg J. I. (2017). Pediatric Stevens-Johnson syndrome and toxic epidermal necrolysis in the United States. J. Am. Acad. Dermatol. 76, 811–817. 10.1016/j.jaad.2016.12.024 28285784 PMC5502094

[B18] Hsu D. Y. Brieva J. Silverberg N. B. Silverberg J. I. (2016). Morbidity and mortality of stevens-johnson syndrome and toxic epidermal necrolysis in United States adults. J. Invest. Dermatol. 136, 1387–1397. 10.1016/j.jid.2016.03.023 27039263

[B19] Hu Y. M. (2001). A case of Stevens-Johnson syndrome due to therapeutic amount of luminal. J. Shanxi Med. Univ. 486. 10.3969/j.issn.1007-6611.2001.06.052

[B20] Huang P.-W. Chiou M.-H. Chien M.-Y. Chen W.-W. Chu C.-Y. (2022). Analysis of severe cutaneous adverse reactions (SCARs) in Taiwan drug-injury relief system: 18-year results. J. Formos. Med. Assoc. 121, 1397–1405. 10.1016/j.jfma.2021.09.025 34674904

[B21] Hung S.-I. Chung W.-H. Liu Z.-S. Chen C.-H. Hsih M.-S. Hui R. C. (2010). Common risk allele in aromatic antiepileptic-drug induced Stevens-Johnson syndrome and toxic epidermal necrolysis in Han Chinese. Pharmacogenomics 11, 349–356. 10.2217/pgs.09.162 20235791

[B22] Jing L. (2018). A case study of Steve Johnson syndrome/toxic epidermal necrolysis induced by antiepileptic drugs. J. Clin. Pharmacother. 16, 78–81. 10.3969/j.issn.1672-3384.2018.09.018

[B23] Kaniwa N. Saito Y. Aihara M. Matsunaga K. Tohkin M. Kurose K. (2008). HLA-B locus in Japanese patients with anti-epileptics and allopurinol-related Stevens-Johnson syndrome and toxic epidermal necrolysis. Pharmacogenomics 9, 1617–1622. 10.2217/14622416.9.11.1617 19018717

[B24] Kaputu-Kalala-Malu C. Ntumba-Tshitenge O. Misson J.-P. (2014). Toxic epidermal necrolysis induced by phenobarbital in a Rwandan child: report of a case. Pan Afr. Med. J. 17, 202. 10.11604/pamj.2014.17.202.3385 25396028 PMC4228990

[B25] Kheir A. E. M. Hamed A. A. (2012). Stevens johnson syndrome secondary to phenobarbitone. Khartoum Med. juornal. Available at: https://www.researchgate.net/publication/277329798_Stevens_Johnson_syndrome_secondary_to_Phenobarbitone.

[B26] Lee S. M. Chung J. Y. Lee Y. M. Park M. S. Namgung R. Park K. I. (2012). Effects of cytochrome P450 (CYP)2C19 polymorphisms on pharmacokinetics of phenobarbital in neonates and infants with seizures. Arch. Dis. Child. 97, 569–572. 10.1136/archdischild-2011-300538 22331680

[B27] Lerch M. Mainetti C. Terziroli Beretta-Piccoli B. Harr T. (2018). Current perspectives on stevens-johnson syndrome and toxic epidermal necrolysis. Clin. Rev. Allerg. Immu. 54, 147–176. 10.1007/s12016-017-8654-z 29188475

[B28] Li X.Na (2013). A case of Stevens-Johnson syndrome induced by intramuscular injection of sodium phenobarbital. Mod. Appl. Pharm. China 30, 109–110. 10.13748/j.cnki.issn1007-7693.2013.01.016

[B29] Man C. B. L. Kwan P. Baum L. Yu E. Lau K. M. Cheng A. S. H. (2007). Association between HLA-B*1502 allele and antiepileptic drug-induced cutaneous reactions in Han Chinese. Epilepsia 48, 1015–1018. 10.1111/j.1528-1167.2007.01022.x 17509004

[B30] Manuyakorn W. Siripool K. Kamchaisatian W. Pakakasama S. Visudtibhan A. Vilaiyuk S. (2013). Phenobarbital-induced severe cutaneous adverse drug reactions are associated with CYP2C19*2 in Thai children. Pediatr. Allergy Immunol. 24, 299–303. 10.1111/pai.12058 23551241

[B31] McCormack M. Farrell J. J. Sills G. J. Kasperavičiūtė D. Carrington M. (2011). HLA-A*3101 and carbamazepine-induced hypersensitivity reactions in Europeans. N. Engl. J. Med. 364, 1134–1143. 10.1056/NEJMoa1013297 21428769 PMC3113609

[B32] McPherson T. Exton L. s. Biswas S. Creamer D. Dziewulski P. Newell L. (2019). British Association of Dermatologists guidelines for the management of StevensJohnson syndrome/toxic epidermal necrolysis in children and young people 2018. Br. J. Dermatol. 181, 37–54. 10.1111/bjd.17841 30829411

[B33] Mockenhaupt M. Messenheimer J. Tennis P. Schlingmann J. (2004). Risk of Stevens–Johnson syndrome and toxic epidermal necrolysis in new users of antiepileptics. Neurology 64, 1134–1138. 10.1212/01.WNL.0000156354.20227.F0 15824335

[B34] Naranjo C. A. Busto U. Sellers E. M. Sandor P. Ruiz I. Roberts E. A. (1981). A method for estimating the probability of adverse drug reactions. Clin. Pharmacol. Ther. 30, 239–245. 10.1038/clpt.1981.154 7249508

[B35] Noe M. H. Micheletti R. G. (2020). Diagnosis and management of Stevens-Johnson syndrome/toxic epidermal necrolysis. Clin. Dermatol. 38, 607–612. 10.1016/j.clindermatol.2020.06.016 33341195

[B36] Page M. J. McKenzie J. E. Bossuyt P. M. Boutron I. Hoffmann T. C. Mulrow C. D. (2021a). The PRISMA 2020 statement: an updated guideline for reporting systematic reviews. BMJ 372, n71. 10.1136/bmj.n71 33782057 PMC8005924

[B37] Page M. J. Moher D. Bossuyt P. M. Boutron I. Hoffmann T. C. Mulrow C. D. (2021b). PRISMA 2020 explanation and elaboration: updated guidance and exemplars for reporting systematic reviews. BMJ 372, n160. 10.1136/bmj.n160 33781993 PMC8005925

[B38] Phillips E. J. Sukasem C. Whirl‐Carrillo M. Müller D. J. Dunnenberger H. M. Chantratita W. (2018). Clinical pharmacogenetics implementation consortium guideline for *HLA* genotype and use of carbamazepine and oxcarbazepine: 2017 update. Clin. Pharmacol. Ther. 103, 574–581. 10.1002/cpt.1004 29392710 PMC5847474

[B39] Qi M. S. (2011). Correlation between CYP2C9 and CYP2C19 gene polymorphisms and phenobarbital blood levels in Hui and Han Chinese patients with epilepsy. J. Stroke Neurol. Dis. 10.19845/j.cnki.zfysjjbzz.2011.09.002

[B40] Qiu Li (2005). A case of paediatric death due to toxic epidermal necrolysis with loosening of the skin caused by phenobarbital. Chin. J. Hosp. Pharm. 483. 10.3321/j.issn:1001-5213.2005.05.060

[B41] Roujeau J.-C. (1994). The spectrum of Stevens-Johnson syndrome and toxic epidermal necrolysis: a clinical classification. J. Invest. Dermatol. 102, 28S–30S. 10.1111/1523-1747.ep12388434 8006430

[B42] Saeed H. Mantagos I. S. Chodosh J. (2016). Complications of Stevens-Johnson syndrome beyond the eye and skin. Burns 42, 20–27. 10.1016/j.burns.2015.03.012 25865527

[B43] Schwartz R. A. McDonough P. H. Lee B. W. (2013). Toxic epidermal necrolysis: Part II. Prognosis, sequelae, diagnosis, differential diagnosis, prevention, and treatment. J. Am. Acad. Dermatology 69, 187.e1–16. 10.1016/j.jaad.2013.05.002 23866879

[B44] Shanbhag S. S. Chodosh J. Fathy C. Goverman J. Mitchell C. Saeed H. N. (2020). Multidisciplinary care in Stevens-Johnson syndrome. Ther. Adv. Chronic Dis. 11, 2040622319894469. 10.1177/2040622319894469 32523661 PMC7236394

[B45] Shi T. Chen H. Huang L. Fan H. Yang D. Zhang D. (2020). Fatal pediatric Stevens–Johnson syndrome/toxic epidermal necrolysis: three case reports. Med. Baltim. 99, e19431. 10.1097/MD.0000000000019431 PMC7220343 32195938

[B46] Shukla S. Rastogi S. Abdi S. A. H. Dhamija P. Kumar V. Kalaiselvan V. (2021). Severe cutaneous adverse reactions in Asians: trends observed in culprit anti-seizure medicines using VigiBase®®. Seizure 91, 332–338. 10.1016/j.seizure.2021.07.011 34274893

[B47] Singh R. K. Rani R. Shankar V. Sinha R. I. (2022). A case of phenobarbitone induced Stevens-Johnson syndrome-toxic epidermal necrolysis along with its causality assessment. Int. J. Basic Clin. Pharmacol. 11, 655. 10.18203/2319-2003.ijbcp20222753

[B48] Sun D. Yu C. Liu Z. He X. Hu J. Wu G. (2014). Association of HLA-B*1502 and *1511 allele with antiepileptic drug-induced Stevens-Johnson syndrome in central China. J. Huazhong Univ. Sci. Technol. Med. Sci. 34, 146–150. 10.1007/s11596-014-1247-7 24496695

[B49] Sun W. Wang J. Ding D. Zhang Q. Wang T. Hong Z. (2019). Blood concentration, efficacy, and adverse events of phenobarbital: a prospective study in rural China. Epilepsy Behav. 90, 247–251. 10.1016/j.yebeh.2018.10.014 30563756

[B50] Trivedi B. Darji N. Malhotra S. Patel P. (2017). Antiepileptic drugs-induced stevens-johnson syndrome: a case series. J. Basic Clin. Pharm. 8, 42–44. 10.4103/0976-0105.195130 PMC5201065 28104975

[B51] Valeyrie-Allanore L. Roujeau J.-C. (2012). “Epidermal necrolysis (Stevens–Johnson syndrome and toxic epidermal necrolysis),” in Fitzpatrick’s Dermatology in General Medicine, 8e. Editors Goldsmith L. A. Katz S. I. Gilchrest B. A. Paller A. S. Leffell D. J. Wolff K. (New York, NY: The McGraw-Hill Companies). Available at: https://accessmedicine.mhmedical.com/content.aspx?bookid=392&sectionid=41138737 (Accessed August 28, 2023).

[B52] Van Nguyen K. Van Vu Q. Tran M. H. Nguyen H. Q. Le C. Q. Dang B. C. T. (2023). Overlapping stevens-johnson syndrome and DRESS syndrome caused by phenobarbital: a Vietnamese case report. Glob. Pediatr. Health 10, 2333794X231216556. 10.1177/2333794X231216556 PMC10699157 38073663

[B53] Wei X. M. (2006). A case of toxic epidermal necrolytic-relaxative drug rash caused by phenobarbital scopolamine. Chin. J. New Drugs, 233–234. 10.3321/j.issn:1003-3734.2006.03.020

[B54] White K. D. Abe R. Ardern-Jones M. Beachkofsky T. Bouchard C. Carleton B. (2018). SJS/TEN 2017: building multidisciplinary networks to drive science and translation. J. Allergy Clin. Immunol. Pract. 6, 38–69. 10.1016/j.jaip.2017.11.023 29310768 PMC5857362

[B55] Yang C.-Y. Dao R.-L. Lee T.-J. Lu C.-W. Yang C.-H. Hung S.-I. (2011). Severe cutaneous adverse reactions to antiepileptic drugs in Asians. Neurology 77, 2025–2033. 10.1212/WNL.0b013e31823b478c 22116946

[B56] Yasiry Z. Shorvon S. D. (2012). How phenobarbital revolutionized epilepsy therapy: the story of phenobarbital therapy in epilepsy in the last 100 years. Epilepsia 53, 26–39. 10.1111/epi.12026 23205960

[B57] Yukawa E. Mamiya K. (2006). Effect of CYP2C19 genetic polymorphism on pharmacokinetics of phenytoin and phenobarbital in Japanese epileptic patients using Non-linear Mixed Effects Model approach. J. Clin. Pharm. Ther. 31, 275–282. 10.1111/j.1365-2710.2006.00712.x 16789993

[B58] Zankat R. Sheth J. G. Chaudhary D. R. D. Malhotra D. S. R. Patel D. P. (2018). A case report on phenobarbitone induced stevens-johnson syndrome: an alarming hypersensitivity reaction. Int. J. Med. Res. Rev. 6, 200–203. 10.17511/ijmrr.2018.i03.12

[B59] Zhang C.Ya (1995). A case of toxic epidermal necrolysis with loosening of the epidermis caused by phenobarbital. J. Bengbu Med. Coll. 305. CNKI:SUN:BANG.0.1995-05-006.

[B60] Zhou J. (2003). Steven-Johnson syndrome induced by phenobarbital. Med. Her. 492. 10.3870/j.issn.1004-0781.2003.07.046

